# Effects of *Saccharomyces cerevisiae* Fermentation on Off-Odour Reduction and Flavour Compounds in Pig Large Intestines

**DOI:** 10.3390/foods14132204

**Published:** 2025-06-23

**Authors:** Ye-Xing Liang, Yun-Cheng Li, Zheng Cao, Xue Li, Ling Zhang, Fan-Bing Meng, Yong-Hua Zhou

**Affiliations:** 1 Agricultural Product Processing Institute, Chongqing Academy of Agricultural Science, Chongqing 401329, China; 18983692739@163.com (Y.-X.L.); snow01241120@163.com (X.L.); jdteazl@163.com (L.Z.); 2 Food Security Publicity and Education Base of Sichuan Province, Chengdu University, Chengdu 610106, China; liyunchengs@126.com (Y.-C.L.); asdf778899zxc@163.com (Z.C.); 3 Xiaojin County Ronggong Zangzhai Food Co., Ltd., Aba Tibetan and Qiang Autonomous Prefecture, Xiaojin County 624299, China; 578883530@163.com

**Keywords:** pig large intestines, *Saccharomyces cerevisiae*, off-odour reduction, fermentation, flavouromics

## Abstract

Pig large intestines (PLIs) are usually processed into various dishes for consumption through cooking methods such as stir frying, stewing, and braising, which are difficult for many consumers to accept because of their unique and pungent off-odours. To reduce the number of off-odour substances present in PLIs, we compared the effects of an untreated control group (blank), added flour (WF), and added *S. cerevisiae* mixed 4% flour in PLIs for fermentation (SC) on the treatment of PLIs. We analysed colour, thiobarbituric acid reactive substance (TBARS) values, and total volatile basic nitrogen (TVB-N) values; additionally, sensory evaluations were performed. The results showed that after 5 h of fermentation, the *S. cerevisiae* mixed flour exhibited the most significant effect on reducing the off-odour of PLIs, exhibited the least effect on the TBARS value and TVB-N was controlled within a reasonable range, while simultaneously maintaining good quality. A total of 415 volatile compounds were identified via flavouromics. Combined with principal component analysis (PCA) and orthogonal partial least squares discriminant analysis (OPLS-DA), the key off-odour substances, including indole (faecal odour), 2-pentylthiophene (fat odour), (E)-2-octenal (fishy odour), and 2-methoxy-phenol (smoky odour), were reduced by 28.1%, 23.90%, 21%, and 22.89%, respectively, after fermentation. Moreover, the content of ethyl octanoate increased 31.04-fold, which enriched the flavour components of the PLIs. The results showed that fermentation of *S. cerevisiae* mixed flour could be used as a method to reduce the off-odours of PLIs.

## 1. Introduction

Pork is the most consumed meat throughout the world and plays an important role in global food culture. According to the statistics of the Food and Agriculture Organization of the United Nations (FAO), the global pork production in 2023 was demonstrated to be 115.5 million tons. Pig large intestines (PLIs) are obtained from the digestive tracts of pigs and are composed of the caecum, colon and rectum; moreover, PLIs are mainly composed of smooth muscle. The average weight of the PLIs in pigs has been observed to be approximately 2.3 kg. In 2023, the global number of live pigs was estimated to be 1.284 billion, and the production of PLIs was approximately 2.9532 million tons, accounting for 0.9~1.8% of the total weight of live pigs. PLIs are large-scale natural food resources [1]. Furthermore, PLIs are rich in fat, protein, vitamins, calcium, phosphorus, and iron, among other nutrients [2]. PLIs are richly abundant in various resources worldwide; however, their processing and utilisation rates are relatively low due to the fact that PLIs represent the part of the digestive tube specialised for the excretion and accumulation of pig faeces. Therefore, fresh PLIs exhibit both a considerable off-odour and pungent odour, which is difficult for consumers to accept [3].

PLIs are suitable for cooking, baking, frying, and barbecuing (among other activities); additionally, they are among the most popular traditional cuisine products in Asia. Dry pot fat sausage, braised fat sausage, stewed fat sausage, and “roasted pig large intestine” are common foods, but they must be cleaned before cooking to reduce odours. Korean institutions typically remove the off-odour by soaking and washing the PLIs with saltwater and white vinegar, as reported by the Korea Food Research Institute. The traditional folk method of reducing the off-odour in PLIs involves the removal of fat oil from PLIs and the addition of flour for cleaning, as well as the removal of the fat and impurities attached to the intestinal wall and subsequent blanching with water; however, the off-odour can continue to be high after treatment, and the effect is not ideal. Boiling water can effectively remove the off-odours of PLIs, thereby representing a common method for reducing the off-odours of PLIs [3]. However, with increasing cooking times, the texture of the PLIs can become soft [2], and the taste can also worsen. Moreover, some producers use laundry detergents or chemical substances that are harmful to humans to remove the off-odours of PLIs [3]. The (E,E)-2,4-undecanonal component of the leaf extract of coriander exhibits a strong deodorising effect on PLIs; however, its extraction cost has been observed to be high [4].

Therefore, a safe, efficient, and economic method to remove off-odours from PLIs is urgently needed. Microbial fermentation is a promising method for reducing off-odours, which has been used to deodorise a variety of foods [5]. This method can transform small molecules of fishy substances (such as aldehydes and ketones) into macromolecular substances lacking flavour due to the action of microbial metabolism; moreover, its metabolic activities produce various flavour substances possessing certain special flavours to achieve the purposes of deodorisation and aroma enhancement. Many studies have demonstrated that *S. cerevisiae* can remove the main fishy substance known as trimethylamine [6]. *S. cerevisiae* fermentation has also been observed to cause significant removal of odour substances in Channa Argus fish head soup and a reduction in the amount of unpleasant volatile components [7].

At present, there are no reports in the literature regarding the application of *S. cerevisiae* for the reduction of the off-odour of PLIs. In this study, when considering the notion that flour was effective in removing the off-odour of PLIs, the addition of *S. cerevisiae* mixed flour for fermentation represented a simple and feasible method that may enhance the odour removal effect. Therefore, we added *S. cerevisiae* mixed flour that was fermented for 1 h, 2 h, 3 h, 4 h, 5 h, and 6 h to determine the optimal odour removal method and time. SPME combined with GC-MS was used for sensory evaluation and volatile flavour component analysis. Additionally, the mechanism of metabolic changes in the main odour substances in PLIs after fermentation with *S. cerevisiae* mixed flour was explored.

Despite the wide availability and nutritional benefits of PLIs, their consumption is limited due to strong and unpleasant odours. Traditional deodorisation methods, such as soaking, boiling, and chemical treatments, often fail to reduce these odours sufficiently and may adversely affect the texture and safety of the product. Microbial fermentation, particularly utilising *S. cerevisiae*, has demonstrated promising deodorising effects in fish-based foods; however, it has not yet been applied to reduce the off-odours of PLIs. Therefore, this study explores the potentiality of using *S. cerevisiae* mixed with flour fermentation as a safe, effective, and low-cost method to reduce off-odours and improve the acceptable consumption of PLIs

## 2. Materials and Methods

### 2.1. Chemicals and Reagents

Fresh PLIs from Duroc pigs (males, aged 6–7 months with weights of 100–120 kg) were purchased from the Chengdu Shiling vegetable market, which had been slaughtered for about 8–10 h. All of the utilised chemical reagents were of analytical grade and were purchased from Shanghai Yuanye Biological Co., Ltd. (Shanghai, China) or Chengdu Cologne Chemicals Co., Ltd. (Chengdu, China). The *S. cerevisiae* strain Saro 47 was isolated from soybean paste starter by the research group, which is currently preserved at College of Food and Biological Engineering, Chengdu University, China. The flavour standard known as 3-hexanone was chromatographically pure and purchased from Sigma-Aldrich Co., Ltd. (St. Louis, MO, USA).

### 2.2. Preparation of Saccharomyces cerevisiae

The *S. cerevisiae* strain was activated in water at 37 °C. Subsequently, 100 µL of the bacterial mixture was inoculated into 100 mL of YPD liquid culture medium, which was subsequently activated and cultured at 30 °C for 24 h. Afterwards, 1 mL of the activated bacterial mixture was inoculated into 400 mL of YPD liquid culture medium at 30 °C for 24 h. The mixture was subsequently centrifuged at 3924× *g* for 10 min at room temperature to obtain bacterial sediment, which was subsequently resuspended in sterile physiological saline to obtain a bacterial suspension.

### 2.3. Samples Preparation

The surface of pig large intestine was washed with flowing water (about 25 °C) until there were no obvious feed remnants and faeces on the surface. The PLIs were then flipped over and the previous step was repeated, and the sample was rubbed twice by hand, each time for 30 s. Then, the excess fat and lymph nodes were removed by scissors. The PLIs were flipped back to their original position. The samples were quartered perpendicularly and weighed. Each sample was of similar weight (approximately 100 g). The grouping of samples is as follows:

(1) The untreated control samples (blank) sample: PLIs without treatment. (2) The flour group (WF) sample: where approximately 100 g PLIs were with the addition of 4% flour by weight of the PLIs; the mixture was fully mixed for 2 min. Then, the sample was cleaned with flowing water until there was no visible flour on its surface. (3) The *S. cerevisiae* mixed flour group (SC) sample: the prepared suspension of *S. cerevisiae* (12.23 × 10^8^ cfu/mL) was added to the PLIs according to the ratio of PLI quality to suspension of *S. cerevisiae* volume of 1:2, and flour (4% by weight of the PLIs) was simultaneously added; subsequently, this mixture was fully mixed for 2 min. Afterwards, the samples were placed into self-sealed zipper bags, and the two layers of the mixture were sealed for fermentation. The fermentation temperature was 30 °C, and the fermentation time was 1 h, 2 h, 3 h, 4 h, 5 h, and 6 h. After fermentation, the sample was cleaned with flowing water until there was no visible flour on its surface. SC1 represented fermentation for 1 h, SC2 represented fermentation for 2 h, SC3 represented fermentation for 3 h, SC4 represented fermentation for 4 h, SC5 represented fermentation for 5 h, and SC6 represented fermentation for 6 h. All the above samples were cooked, chopped into minced meat, and manually homogenised for 2 min. The samples were subsequently vacuum packed and refrigerated at −80 °C for further analysis.

### 2.4. Determination of Colour

The colours of the PLI samples were measured via an NH300 portable colorimeter (China Sanlian Technology Co., Ltd., Xi’an, China). Six measurements were performed at different positions on the sample, and the values of L*, a*, and b* were separately recorded.

### 2.5. Determination of the pH Value

The pH values of the PLI samples were measured using a Testo 205 portable pH meter (Testo SE & Co. KGaA., Schwarzwald, Germany), and the measurement values at three different positions on the samples were recorded.

### 2.6. Determination of the TBARS Value

The TBARS values of the samples were determined according to the methods of Chang [8] and Xiong [9], with slight modifications. Five grams of chopped sample was blended with 50 mL of trichloroacetic acid (TCA) mixture. The mixture was then shaken at 50 °C for 30 min on a THZ-82 constant temperature shaker (Changzhou Aohua Instrument Co., Ltd., Changzhou, China) and cooled to room temperature. The mixture was centrifuged at 6000 rpm for 10 min, after which 5 mL of the supernatant was mixed with 5 mL of thiobarbituric acid (TBA); subsequently, the mixture was reacted in a 90 °C water bath in a 15 mL centrifuge tube for 30 min. A mixture of trichloroacetic acid and thiobarbituric acid was used as a blank measurement, and the absorbance was determined at 532 nm with a UV–Vis spectrophotometer (Shanghai Yuanxi Instrument Co., Ltd., Shanghai, China). The calculation formula was as follows (1):

(1) $$TBARS\;(mg/kg)=\frac{A_{532}}{n}\times 9.48$$

where n represents the sample mass.

### 2.7. Determination of the TVB-N Value

The TVB-N values of the samples were measured according to the method described by Fu [10], with slight modifications. One hundred millilitres of pure water was added to 20 g of chopped sample, after which the mixture was magnetically stirred to evenly disperse the sample in the solution; afterwards, the mixture was allowed to stand for 30 min, after which it was filtered to obtain the filtrate. A total of 10 mL of the filtrate was collected and measured with a KDN-102C Kjeldahl nitrogen analyser (Shanghai Qianjian Instrument Co., Ltd., Shanghai, China). The TVB-N value was determined based on the consumption of hydrochloric acid, and the calculation formula was as follows (2):

(2) $$\text{TVB-N}\;(mg/100\;g)=\frac{\left(V_{1}-V_{2}\right)\times C\times 14}{m\times \frac{V}{V_{0}}}\times 100$$

where V_1_ is the amount of titrated hydrochloric acid, V_2_ is the amount of hydrochloric acid titration of the blank sample, C is the concentration of hydrochloric acid, m is the mass of the sample, V is the volume of the filtrate, and V_0_ is the total volume of the sample mixture.

### 2.8. Sensory Evaluation

The sensory evaluation was performed according to Li et al.’s method [2], with some modifications. Well-trained evaluators (5 males and 5 females) conducted sensory evaluations of the PLI samples. For the evaluation, 5.0 g of each sample was randomly placed in a 10 mL bottle. The samples were evaluated every 5 min, and the evaluators rinsed their nasal passages between the tests. The evaluation was conducted in a professional laboratory at 20 °C. The specific scoring criteria are shown in Table 1.

### 2.9. SPME Processing and Volatile Substance Analysis

The PLI samples were ground with liquid nitrogen and evenly vortexed, and 0.2 g of each sample was weighed into a headspace bottle. Afterwards, 0.2 g of NaCl and 20 μL (10 μg/mL) of internal standard solution were added, and the samples were extracted by HS-SPME. The samples were analysed by GC-MS (8890-7000E, Agilent, Inc., Santa Clara, CA, USA).

The following GC conditions were utilised: DB-5MS capillary column (30 m × 0.25 mm × 0.25 μm), high-purity He gas used as the carrier gas (>99.99%), flow rate of 1.2 mL/min, inlet temperature of 250 °C, and solvent delay of 3.5 min. The initial column temperature was maintained at 40 °C for 3.5 min, increased to 100 °C at 10 °C/min, increased to 180 °C at 7 °C/min, and finally increased to 280 °C at 25 °C/min and maintained for 5 min.

The following MS conditions were used: electron bombardment ion source (EI), ion source temperature of 230 °C, quadrupole temperature of 150 °C, mass spectrometry interface temperature of 280 °C, and electron energy of 70 EV. The utilised scanning mode was the ion detection mode (SIM), and accurate qualitative and quantitative ion scanning were performed.

For the qualitative analysis, compounds were identified via their mass spectra by searching the database. This tool was independently established based on National Institute of Standards (NIST) and Willey spectrum libraries. The retention indices (RIs) of the volatile flavour compounds were calculated based on the retention times of normal alkanes, and the flavour substances included volatiles to be measured by matching with the standard compounds.

For the quantitative analysis, 3-hexanone was added to the samples as the internal standard, and the tested component and internal standard were simultaneously detected. The relative contents of the volatile flavour substances in the samples were calculated according to the principle that the peak area of the compound was proportional to the content, and the calculation method was as follows (3):

(3) $$X_{i}=\frac{V_{s}\times C_{s}}{M}\times \frac{I_{i}}{I_{s}}\times 10^{-3}$$

where X_i_ is the content of the compound being measured (μg/g); V_s_ is the volume of the internal standard (μL); C_s_ is the internal standard mass concentration (μg/mL); M is the mass of the sample to be measured (g); I_s_ is the peak area of the internal standard; and I_i_ is the peak area of Compound i in the sample to be measured.

### 2.10. Statistical Analysis

The unsupervised measurement tool was used for principal component analysis, the MS spectrum Pattern software (Puxi Inc., Beijing, China) was used for heatmap analysis, and OPLS-DA was used for R (metaboanalystr1.0.1) analysis. The data are expressed as the means ± standard deviations (SDs), which were considered to be statistically significant at a *p* value < 0.05. All experiments were performed in triplicate with 3 observations. Statistical analysis for the PLIs with different treatments was performed using one-way analysis of variance (ANOVA), and significant differences were identified using Duncan’s test. IBM SPSS statistics version 20.0 was used for the statistical calculations and analysis.

## 3. Results and Discussion

### 3.1. Colours of the PLIs Under Different Treatments

The colours of PLIs directly affect consumers’ purchasing decisions. Additionally, the colours of PLIs represent an important standard for determining the freshness and cleanliness of the PLIs. High-quality PLIs should ideally be light yellow or milky white with smooth surfaces and no abnormal spots. The value of L* is positively correlated with brightness. Moreover, positive values of a* and b* represent red and yellow colours, respectively, whereas negative values of a* and b* represent green and blue colours, respectively [11]. As shown in Table 2, the SC group values of L* of the PLIs were not significantly different from those of the WF or blank samples, thereby indicating that flour treatment demonstrated little effect on the brightness of the PLIs. However, the L* values of the PLIs in the SC group tended to initially decrease, after which they increased and subsequently decreased again, reaching a maximum value of 74.55 in SC5; moreover, those values in the blank and WF groups did not significantly differ. The a* value reached a maximum value of 5.04 at SC5, which was significantly different from those maximum values of the blank and WF groups. Moreover, the b* value initially increased and subsequently decreased, with the value of SC5 reaching a maximum value of 13.97, which was significantly different from those values of the blank and WF groups. This finding revealed that *S. cerevisiae* fermentation exhibited no significant effect on the brightness of the PLIs, whereas the red and yellow colours of the PLIs significantly increased, which more closely matched the colours of high-quality PLIs. These colours lead people to believe that the PLIs are fresher and that the sensory quality is improved. Therefore, the effect of fermentation with *S. cerevisiae* mixed flour for 5 h was greater than sole treatment with flour.

### 3.2. pH Values of PLIs Under Different Treatments

**Figure 1:**
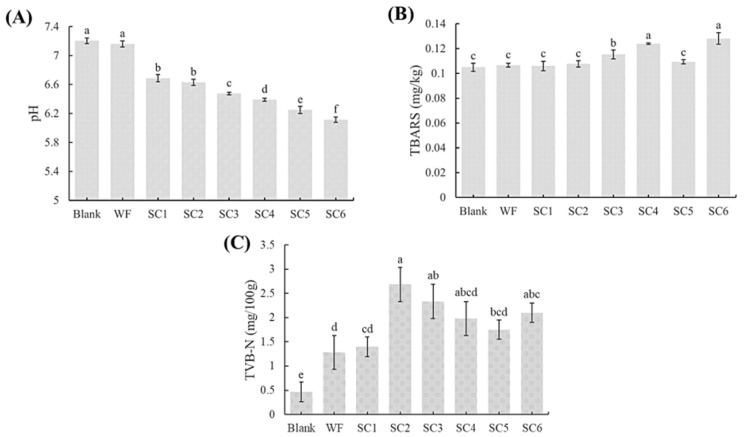
(**A**) pH values of the PLIs produced via different processing methods. (**B**) TBARS values of PLIs processed via different processing methods. (**C**) TVB-N values of PLIs under different processing methods. Values with different lowercase letters in the same figure are significantly different (*p* < 0.05).

A reduction in pH can inhibit the growth of microorganisms and help to extend the shelf-life of food [12]. As shown in Figure 1A, there was no significant difference observed in the pH value between the WF and blank groups. The pH value of the PLIs in the SC group decreased over time, which was significantly different from that in the WF group and the blank group. The decrease in pH may have been a result of the accumulation of organic acids during fermentation [13]. PLIs and flour contain certain amounts of sugar and fat, which provide substrates for the growth and metabolism of *S. cerevisiae*. When these substrates are utilised by *S. cerevisiae*, the accumulation of organic acids leads to a decrease in pH. Adesulu-Dahunsi et al. reported that *S. cerevisiae* fermentation can reduce the pH value of meat used in sauce and subsequently control the growth of spoilage bacteria in this meat, thereby increasing the ease of storage [14]. However, as the fermentation time increased, the accumulation of organic acids affected the flavour of the PLIs.

### 3.3. TBARS Values of PLIs Under Different Treatments

The TBARS value reflects the content of malondialdehyde compounds formed by lipid oxidation, which can reflect the degree of oxidative deterioration of the fat in food [15]. As shown in Figure 1B, there was no significant difference observed in TBARS values between the WF and blank groups, thus indicating that flour treatment of PLIs did not affect fat oxidation. The TBARS value of the SC group initially increased, after which it decreased and subsequently increased again during fermentation. During the SC1-SC4 stages, the lipid oxidation rate was accelerated due to the active growth and metabolic activities of *S. cerevisiae*. However, during the SC4-SC5 stages, *S. cerevisiae* may produce several antioxidant substances that can capture free radicals or reactive oxygen species, thereby reducing the occurrence of lipid peroxidation; thus, there was no significant difference observed between the SC5 group and the blank group. During the SC5–SC6 stages, with increasing fermentation time and further changes in fermentation conditions, the metabolic activity of *S. cerevisiae* gradually decreased, and the production of antioxidant substances correspondingly decreased, thereby leading to another increase in the TBARS value. Therefore, fermentation for 5 h exhibited the least effect on the TBARS value of the sample, thus resulting in the lowest degree of lipid oxidation. These findings indicate that fermentation with *S. cerevisiae* mixed flour demonstrates a certain antioxidant effect and can prevent the excessive oxidation of food from producing off-odours [16].

### 3.4. TVB-N Values of PLIs Under Different Treatments

A higher TVB-N value corresponds to a greater degree of protein and amine decomposition, which is often used as an indicator of meat freshness [17]. As shown in Figure 1C, the TVB-N value of the SC group tended to initially increase, after which it decreased and subsequently increased again during fermentation. At the SC1–SC2 stages, the increase in the TVB-N value may be due to the degradation of protein and other nitrogen-containing compounds caused by protein oxidation during the fermentation process, which led to the accumulation of organic amines and a corresponding increase in the TVB-N value [18]. At the SC2–SC5 stages, the decrease in the TVB-N value may be due to the production of antioxidant substances by fermentation, which can reduce the oxidation and decomposition of proteins; additionally, there was a significant difference observed between the SC5 and SC2 stages. At the SC6 stage, the TVB-N value increased again, thereby indicating that the production of antioxidant substances decreased, which corresponded to an increase in the TVB-N value. Fresh and frozen lean pork should contain less than 15 mg of TVB-N per 100 g according to the National Standards of the People’s Republic of China (GB/T 9956.2-2008 [19]). Although fermentation slightly reduced the freshness of PLIs, TVB-N was still controlled within a reasonable range, and the fermentation time was controlled within 5 h to avoid a further reduction in the freshness of PLIs with prolonged fermentation time.

### 3.5. Sensory Evaluation of PLIs Under Different Treatments

**Figure 2:**
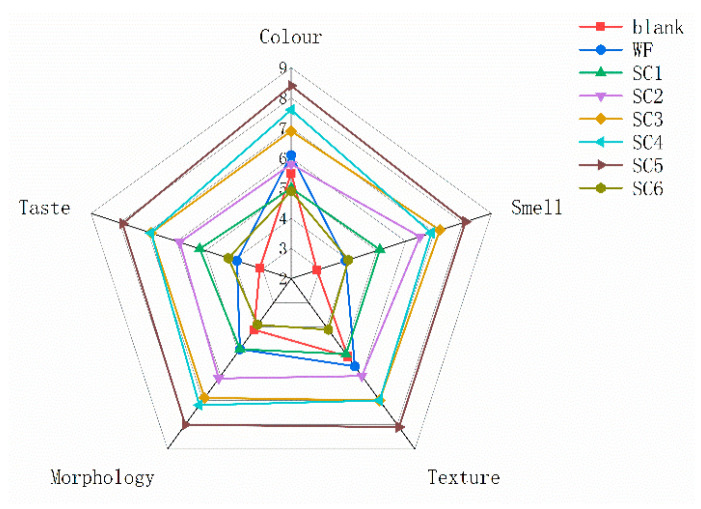
Sensory score of PLIs under different processing methods.

Sensory indicators are particularly important in the food industry and can directly affect consumers’ purchasing decisions and satisfaction. As shown in Figure 2 and Table S1, the sensory score of the WF group was greater than that of the blank group, and the sensory score of the SC group gradually increased with increasing fermentation time, reaching the maximum value at SC5. The sensory score of the blank group was the lowest, with obvious off-odour such as faecal smell and fishy smell. After fermentation for 5 h, the odour significantly decreased and the sensory score increased. However, when fermented for 6 h, the taste, texture, and smell of PLIs significantly decreased, and sensory scores significantly decreased, which was consistent with the TBARS results. The sensory quality of PLIs improved with prolonged fermentation time because fermentation may have consumed off-odour substances and produced new aroma substances. The sensory improvement effect became more pronounced with prolonged fermentation time, especially after 5 h of fermentation.

In conclusion, compared with those of the blank and WF samples and with respect to SC5, the values of a* and b* of the PLIs significantly increased, the TBARS value did not significantly change, and a maximum sensory score was achieved. Therefore, the best method for removing off-odours from PLIs is to ferment *S. cerevisiae* mixed flour for 5 h. Subsequently, we further compared and analysed the volatile flavour compounds of the SC5, blank, and WF groups to verify the effects of odour removal.

### 3.6. Flavouromics Analysis of PLIs Under Different Treatments

#### 3.6.1. PCA

**Figure 3:**
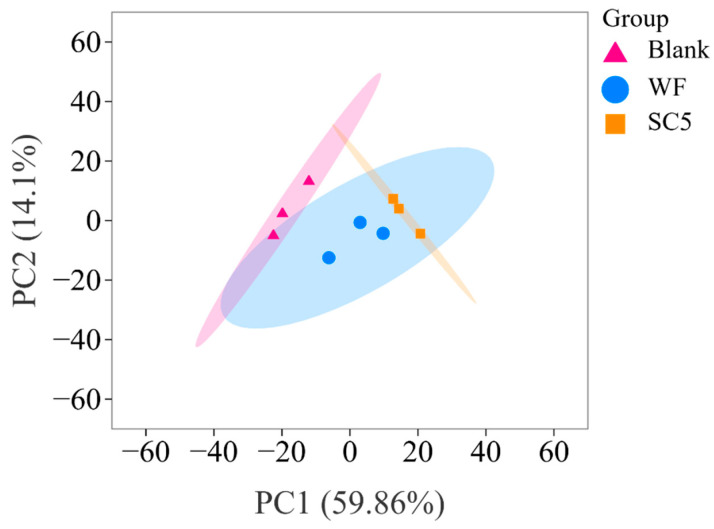
Diagram of volatile metabolites.

PCA was conducted to analyse the differences in volatile metabolites in PLIs under different treatments. As shown in Figure 3, the overall variability of the principal components known as PC1 and PC2 was 73.96%, and PC1 and PC2 explained 59.86% and 14.1% of the overall variance, respectively. The distance between different colour points in the PCA diagram indicates the similarities or differences observed among the sample groups [1]. The colour points of the three sample groups were relatively dispersed, and the distance between SC5 and WF was relatively smaller, thereby indicating that they had relatively similar aroma profiles. However, the distance between SC5 and the blank group was greater, thus suggesting that the aroma profile of SC5 was significantly different from that of the blank group. It showed that the flavour compounds in the sample change after the *S. cerevisiae* fermentation mixed flour treatment, which was consistent with the results of sensory evaluation.

#### 3.6.2. Orthogonal Partial Least Squares Discriminant Analysis (OPLS-DA)

**Figure 4:**
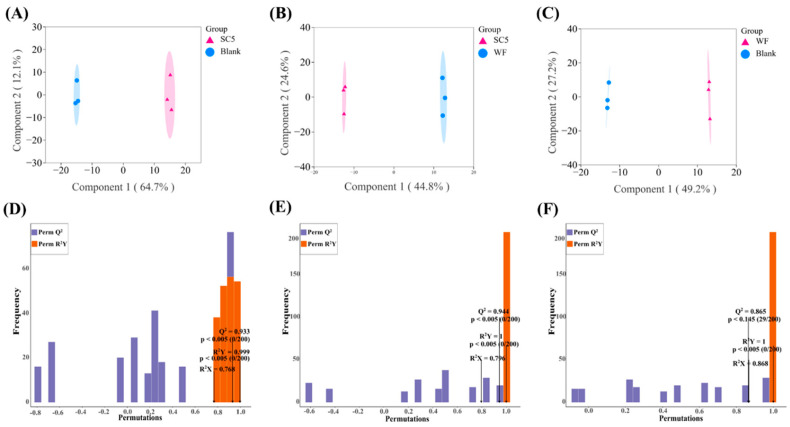
Plots of OPLS-DA scores for two-by-two comparisons (**A**–**C**) and their model validation plots (**D**–**F**). (**A**,**D**) SC5 vs. blank; (**B**,**E**) SC5 vs. WF; (**C**,**F**) WF vs. blank.

OPLS-DA was utilised to confirm the correlation between the identified key volatiles and to establish a discriminant model [20]. OPLS-DA distinguishes samples via an orthogonal signal correction filter [21]. As shown in Figure 4A–C, the OPLS-DA score chart clearly revealed separation between SC5 and the blank group, SC5 and the WF group, and the WF and blank groups, thereby indicating that the volatile components of the PLIs significantly changed. The OPLS-DA model was used to analyse the metabolome data and draw a score map of each group, in order to demonstrate the differences between each group [22], where Q^2^ represents the prediction ability and R^2^X and R^2^Y represent the interpretation rates of the X and Y matrices, respectively. The closer the R^2^Y and Q^2^ values are to 1, the stronger the reliability and predictive ability of the model. Figure 4D–F show that the Q^2^, R^2^X, and R^2^Y values of the SC5 samples vs. the blank samples were 0.933, 0.768, and 0.999, respectively. Moreover, the Q^2^, R^2^X, and R^2^Y values of the SC5 samples vs. the WF samples were 0.944, 0.796, and 1, respectively, and the Q^2^, R^2^X, and R^2^Y values of the WF samples vs. the blank samples were 0.865, 0.868, and 1, respectively, thereby indicating that the model was stable and reliable.

#### 3.6.3. Volatile Compounds of PLIs Under Different Treatments

**Figure 5:**
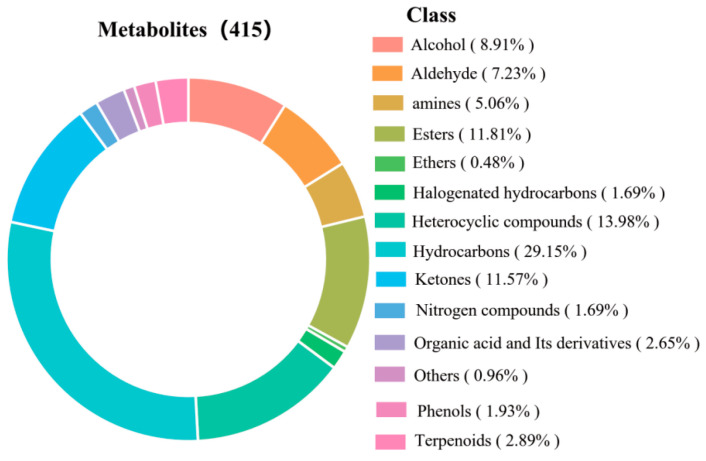
Classification map of the total amount of volatile metabolites.

Flavour is one of the most important factors influencing consumers’ preferences for processed meat products [23]. As shown in Figure 5, a total of 415 volatile substances were identified in the PLIs, which could be classified into 14 categories, including 37 alcohols, 30 aldehydes, 21 amines, 49 esters, 2 ethers, 7 halogenated hydrocarbons, 58 heterocyclic compounds, 121 hydrocarbons, 48 ketones, 7 nitrogen compounds, 11 organic acids (and their derivatives), 8 phenols, 12 terpenoids, and 4 other substances. The most abundant volatiles included hydrocarbons (29.15%), heterocyclic compounds (13.98%), esters (11.81%), and ketones (11.57%).

#### 3.6.4. Identification and Analysis of Differentially Abundant Metabolites

The differentially abundant metabolites were selected according to the conditions of VIP > 1 and *p* < 0.05. In the comparison of the SC5 and blank samples, 214 volatile metabolites were observed to be significantly different, of which 213 metabolites were significantly downregulated, and 1 metabolite was significantly upregulated. In the comparison of the SC5 and WF samples, 24 significantly different metabolites were detected, including 23 significantly downregulated metabolites and 1 significantly upregulated metabolite. A comparison of the WF and blank samples revealed 61 significantly different metabolites, which were all observed to be significantly downregulated. According to the conditions of FC > 1.5 or <0.67, the top 50 differentially abundant metabolites were selected, which mainly included heterocyclic compounds, aldehydes, ketones, esters, hydrocarbons, amines, alcohols, and phenols. Changes in these volatile metabolites can affect the flavour of PLIs.

##### Heterocyclic Compounds

**Figure 6:**
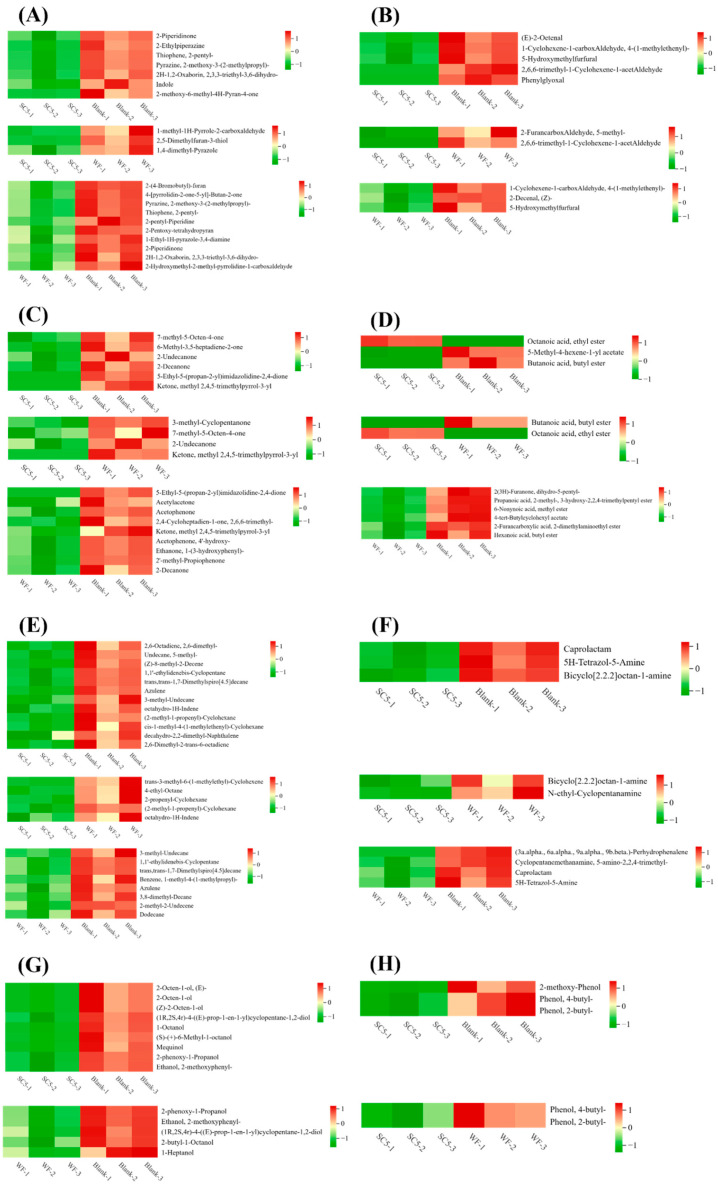
Heatmap of differentially volatile metabolites. (**A**) Heterocyclic Compounds; (**B**) Aldehydes; (**C**) Ketones; (**D**) Esters; (**E**) Hydrocarbons; (**F**) Amines; (**G**) Alcohols; (**H**) Phenols.

Heterocyclic compounds are important flavour substances of meat [24], and are mostly derived from the thermal oxidative degradation of protein and thiamine [25]. As shown in Figure 6A, the contents of 2,3,3-triethyl-3,6-dihydro-2H-1,2-oxaborin, 2-ethylpiperazine, 2-methoxy-6-methyl-4H-pyran-4-one, 2-piperidinone, 2-pentylthiophene, 2-methoxy-3-(2-methylpropy)-pyrazine, and indole were significantly lower in the SC5 samples compared to the blank samples. Indole, an imine substance that derives from the metabolism of tryptophan by gut microflora, is a characteristic volatile substance that causes the faecal odour of PLIs [2,26,27], which was reduced by 28.1%. It indicated that *S. cerevisiae* may inhibit the production of off-odour substances by gut microflora. Studies have shown that tryptophan degraded by the microbials was considered to promote the formation of aromatic compounds. Indole metabolites were assumed precursors of other aromatic substances. Zhang et al. speculated that tryptophan metabolism was the metabolic pathway that affected the flavour substances of dough fermented *by S. cerevisiae* or by *S. cerevisiae* and *L. plantarum* [28]. Therefore, the reduction of indole in the PLIs during fermentation by *S. cerevisiae* might be attributed to the improvement of flavour through tryptophan metabolism. Moreover, 2-pentylthiophene is a sulphur-containing compound with a fat odour (which may be generated by thiamine or cysteine carbon) [29], which was reduced by 23.90% in the SC5 samples. The results demonstrated that fermentation with *S. cerevisiae* mixed flour could effectively reduce the characteristic off-odour substances in PLIs and reduce the decomposition of fat and protein. Compared with those of WF samples, the contents of 1-methyl-1H-pyrrole-2-carboxaldehyde, 2,5-dimethylfuran-3-thiol, and 1,4-dimethylpyrazole in the SC5 samples were significantly decreased.

The decrease in indole content indicated that indole was consumed in metabolism. Indole is an imine substance, which may have an unpleasant taste at high concentrations.

##### Aldehydes

Aldehydes are produced by lipid oxidation and thermal degradation [30]. Due to their low odour threshold, they are generally considered to be the main flavour contributors to fermented beverages [31]. As shown in Figure 6B, the contents of (E)-2-octenal, 5-hydroxymethylfurfural, 4-(1-methylethenyl)-1-cyclohexene-1-arboxaldehyde, 2,6,6-trimethyl-1-cyclohexene-1-acetaldehyde, and phenylglyoxal were significantly lower in the SC5 samples compared to the blank samples. It indicated that fermentation may have reduced aldehydes, and this was consistent with the findings of Gao et al. Aldehydes were known to be the major contributors to off-odours in fish products, and the fermentation treatment using yeast was very effective in diminishing negative volatile compounds to the flavour of the fish head soup [7]. Moreover, (E)-2-octenal is the main odour compound detected in the porcupine liver [26]. Additionally, it is a key fishy odour in aquatic products, which can produce unpleasant and pungent odours [32], which was reduced by 21% in SC5 samples. The reduction in aldehydes may be related the enzymes produced by microbial fermentation. Nedele et al. reported that diverse biopathways in different species of microorganisms were hypothesised, but the key enzyme seemed to be aldehyde dehydrogenase oxidising the aldehydes into corresponding alcohols [33]. Qiu et al. reported that the lactic acid bacteria fermentation process promoted the reduction of aldehydes, thereby resulting in better aroma properties in fermented samples than in unfermented samples [31]. (E)-2-nonenal and (E)-2-octenal showed undesirable odours such as grassy and fatty odours, which would be defined as having a “beany flavour”. It was inferred that the reduction of that might be related to alcohol dehydrogenase in *S. cerevisiae*. During yeast metabolism, the action of alcohol dehydrogenase reduced aldehydes to their corresponding alcohols, thereby improving the overall odour characteristics. There were two different reduction pathways for monounsaturated aldehydes (exemplified by the degradation of (E)-2-nonenal): (i) it was reduced to (E)-nonanol, which was then reduced to nonanol; (ii) its α, β-unsaturated double bond was hydrogenated to form nonanal, which was then reduced to nonanol by alcohol dehydrogenase [34,35].

##### Ketones

Ketones mainly originate from polyunsaturated fatty acid metabolism or alcohol oxidation and exhibit strong flavours at low concentrations [36]. As shown in Figure 6C, via the comparison of SC5 and blank samples, six types of ketones were significantly reduced in the SC5 samples. Ketones typically have fruity, sweet, herbal, and creamy aromas [37,38]. The decrease in ketone content may be due to the continuous decrease in pH during the fermentation process of PLIs, thereby affecting the stability of ketones (which are oxidised to carboxylic acids) [38]; conversely, this decrease may be related to the adsorption effect of flour. Yen et al. reported that C_4_–C_11_ ketones or volatiles develop green, fatty, metallic, and dirt aromas that synergistically contribute to the characteristic fishy flavour [39]. The kelp fermented with *S. cerevisiae* showed a decrease in fishy odours, which could potentially be associated with the enzymatic activity of enone reductase in yeast, catalyzing the conversion of unsaturated carbonyl compounds into substances with higher odour thresholds [40]. This might be the reason for the downregulation of unsaturated ketone substances such as 6-methyl-2,5-hepten-2-one.

##### Esters

Esters are usually derived from the esterification or enzymatic reaction of fatty alcohols and free fatty acids; moreover, ethyl butyrate and ethyl caproate exhibit fruity aromas and can reduce the degree of irritation caused by fatty acids or amines [41]. As shown in Figure 6D, via a comparison between the SC5 and blank samples, the contents of 5-methyl-4-hexen-1-yl acetate and butanoic acid butyl ester were significantly decreased in the SC5 samples, whereas the content of octanoic acid ethyl ester was significantly increased in the SC5 samples. Octanoic acid ethyl ester has a sweet, fruity apple–banana flavour, which is among the key flavours of Chinese Moutai liquor [42], and may mask the off-odour of PLIs; the ester was increased 31.04-fold in the SC5 samples. A comparison of the SC5 and WF samples revealed that the content of octanoic acid ethyl ester was increased 26.38-fold in the SC5 samples. Amino acid metabolism is an important mechanism in the production of aroma compounds by *S. cerevisiae* fermentation. Lu et al. reported that amino acid addition in a simulated juice system could promote octanoic acid synthesis during fermentation by *S. cerevisiae.* The addition of amino acids affected the metabolic pathway of pyruvate to alcohols, acids and esters [43]. Therefore, we speculate that the increase in octanoic acid ethyl ester may be due to the amino acid metabolism during fermentation, which are then converted to octanoic acid under the action of *S. cerevisiae*; octanoic acid then reacts with ethanol to produce octanoic acid ethyl ester, and thus, increasing its content can contribute to the positive changes in the odour pattern of PLIs.

##### Hydrocarbons

Hydrocarbons are mainly produced by lipid decomposition. Qi et al. [44] reported that the sensory threshold of hydrocarbons is high and that their impact on the aroma perception of chicken soup is limited. As shown in Figure 6E, via the comparison of the SC5 and blank samples, 12 types of hydrocarbons were significantly decreased in the SC5 samples. Azulene is a bicyclic nonbenzene aromatic hydrocarbon that is found in Chinese herbal plants (such as green grass) and has a pungent flavour [45]; the content of this hydrocarbon was decreased by 24%.

##### Amines

Amines usually produce pungent odours, special off-odours, and fishy odours [32]. As shown in Figure 6F, via the comparison of the SC5 and blank samples, the contents of 5H-tetrazol-5-amine, bicyclo[2.2.2]octane-1-amine, and caprolactam were significantly reduced by 23.72%, 23.91%, and 23.76%, respectively, in the SC5 samples. Caprolactam is one of the five most abundant volatiles detected in the faeces of Przewalski’s horses [46]. A comparison of the SC5 and WF samples revealed that the contents of bicyclo[2.2.2]octane-1-amine and N-ethyl-cyclopentamine were significantly decreased by 11.72% and 15.69%, respectively, in the SC5 samples. The *S. cerevisiae* HL10 and HL17 were able to completely degrade histamine and tyramine within 24 h, which might be attributed to the activity of amine oxidases [47]. The reduction in amines may reduce the off-odour of PLIs and improve their flavour, thereby indicating that fermentation of *S. cerevisiae* mixed flour can reduce the off-odour of PLIs.

##### Alcohols

Alcohols are typically derived from the degradation and oxidation of lipids. As shown in Figure 6G, nine types of alcohols were significantly decreased in the SC5 samples compared with the blank samples. (E)-2-octen-1-ol, (Z)-2-octyl-1-ol, 1-octanol, (s)-(+)-6-methyl-1-octanol, mequinol, and 2-methoxyphenyle-thanol are higher alcohols, which were observed to be decreased by 19.19%, 19.19%, 19.73%, 22.10%, 22.89%, and 23.69%, respectively, in the SC5 samples. Higher alcohols decompose in the human body at slow rates. Additionally, higher alcohols exhibit a certain flavour; however, excessive consumption of higher alcohols is harmful to the human. For example, the higher alcohol content in kiwifruit wine is often excessive, posing the risk of toxic effects on the health of drinkers. Thus, it is necessary to control the content of higher alcohols in fruit wines [48]. Guymon et al. [49] reported that higher alcohols are mainly synthesised by yeast from monosaccharides (the Harris pathway) and from branched chain amino acids (the Ehrlich pathway) during alcohol fermentation, with approximately 75% of higher alcohols being produced through the Harris pathway. The decrease in the content of higher alcohols may be due to changes in the metabolic pathway of yeast during fermentation. *S. cerevisiae* utilises monosaccharides in flour to synthesise alcohols, which subsequently react to produce esters, thereby reducing the production of higher alcohols.

##### Phenols

Phenols are primarily aroma compounds detected in smoked food [50]. As shown in Figure 6H, via the comparison of SC5 and blank samples, the contents of 4-butyl-phenol, 2-butyl-phenol, and 2-methoxy-phenol were significantly decreased by 52.35%, 52.35%, and 22.89%, respectively, in the SC5 samples. 2-methoxy-phenol has been observed to exhibit a smoky taste [50]. When comparing the SC5 and WF samples, the contents of 4-butyl-phenol and 2-butyl-phenol were decreased by 36.85% in the SC5 samples. 4-methylphenol have been detected in animal fat and have lipophilic properties (accumulating in fat tissue). 4-methylphenol produced by gut microflora are characteristic off-odour substances of PLIs, which could be reduced by cooking treatments [1,2]. Multiple enzymes are involved in the oxidation, degradation, hydroxylation, methylation, and epimerisation of phenols. Polyphenol oxidase can oxidise phenols into quinones. The ethanol dehydrogenase transfers active hydrogen atoms to the coenzyme NAD or NADP and oxidises the phenolic groups of phenols into aldehyde or ketone groups [51]. The reduction of phenols may be related to oxidation and degradation caused by the fermentation of *S. cerevisiae*. These reduced phenols may help to reduce the off-odour of PLIs and better meet the taste preferences of consumers.

## 4. Conclusions

In this study, *S. cerevisiae* and flour were added to PLIs for fermentation in order to reduce the off-odour of PLIs. The results revealed that PLIs via *S. cerevisiae* mixed flour fermentation 5 h maintained a good level of freshness, with the best sensory quality and effect of reducing the off-odour. To verify the effect of odour removal, the SC5 samples were subsequently compared with WF and blank samples for flavouromics analysis. The key off-odour substances of PLIs, such as indole, 2-pentylthiophene, (E)-2-octenal, and 2-methoxy-phenol, were reduced significantly, respectively, in the SC5 samples compared with those of the blank samples, which could be the main reasons explaining the reduction in the off-odour of PLIs. Furthermore, the content of octanoic acid ethyl ester was increased significantly, which enriched the flavour components of the PLIs after fermentation. In summary, the removal of the off-odour by the *S. cerevisiae* fermentation mixed flour treatment for 5 h was better than that due to the flour treatment alone. The *S. cerevisiae* mixed flour fermentation may be applied in the food processing practices of PLIs. Furthermore, future studies are needed to optimise the process for industrial use or evaluate consumer acceptance.

## Figures and Tables

**Table 1:** Sensory grading scale for PLIs.

Items	Standards of Grading	Score
Colour	The colour is uniform with lustre	8~10
	The colour is too dark or too light with slight lustre	5~7
	The colour is uneven with mottled and poor gloss	1~4
Smell	The smell is mellow and lacks an off-odour smell, along with a slight wine aroma	8~10
	The smell is moderate with slightly off-odours of the PLIs, along with a light wine aroma	5~7
	The smell has obvious off-odours of the PLIs without a wine aroma	1~4
Texture	Elasticity is excellent, and the texture is layered	8~10
	Elasticity is moderate, and the texture is good	5~7
	The texture is very hard and inelastic	1~4
Morphology	The structure of the tissue morphology is complete and is not sticky	8~10
	The structure of the tissue morphology is relatively complete and is slightly sticky	5~7
	The structure of the tissue morphology is loose and has a sticky surface	1~4
Taste	The taste is coordinated, and the flavour of the PLIs is well combined with the fermentation flavour	8~10
	The taste is general, and the integration of the flavour of the PLIs with the flavour of the fermentation is insufficient	5~7
	The taste is poor, and the flavour of the PLIs and the flavour of the fermentation are not integrated	1~4

**Table 2:** Colour changes in PLIs under different processing methods.

Samples	L*	a*	b*
Blank	72.24 ± 0.95 ^ab^	2.09 ± 0.19 ^b^	11.25 ± 0.54 ^cd^
WF	74.30 ± 2.05 ^a^	2.53 ± 1.63 ^b^	10.77 ± 0.90 ^d^
SC1	68.26 ± 3.92 ^b^	4.64 ± 0.21 ^a^	12.15 ± 0.03 ^bc^
SC2	70.76 ± 1.96 ^ab^	4.39 ± 0.10 ^a^	12.53 ± 0.38 ^b^
SC3	72.40 ± 2.12 ^ab^	4.56 ± 0.11 ^a^	12.98 ± 0.65 ^ab^
SC4	73.21 ± 3.89 ^a^	5.03 ± 0.31 ^a^	12.54 ± 0.65 ^b^
SC5	74.55 ± 1.25 ^a^	5.04 ± 0.10 ^a^	13.97 ± 0.53 ^a^
SC6	70.32 ± 1.39 ^ab^	4.51 ± 0.92 ^a^	13.09 ± 0.36 ^ab^

Values with different lowercase letters in the same column are significantly different (*p* < 0.05). The blank group represents untreated control samples. The WF group represents PLI samples with the addition of 4% flour by weight of the PLIs. The SC group represents PLI samples fermented with S. cerevisiae and 4% flour, SC1 represented fermentation for 1 h, SC2 represented fermentation for 2 h, SC3 represented fermentation for 3 h, SC4 represented fermentation for 4 h, SC5 represented fermentation for 5 h, SC6 represented fermentation for 6 h.

## Data Availability

The original contributions presented in this study are included in the article/Supplementary Materials. Further inquiries can be directed to the corresponding author.

## Supplementary Materials

The following supporting information can be downloaded at: https://www.mdpi.com/article/10.3390/foods14132204/s1, Table S1. All flavor substance data in PLIs under different processing methods. Table S2. The flavor substance data between SC5 and blank. Table S3. The flavor substance data between SC5 and WF. Table S4.The flavor substance data between WF and blank.
